# Strategies to Inhibit Myc and Their Clinical Applicability

**DOI:** 10.3389/fcell.2017.00010

**Published:** 2017-02-23

**Authors:** Jonathan R. Whitfield, Marie-Eve Beaulieu, Laura Soucek

**Affiliations:** ^1^Vall d'Hebron Institute of Oncology, Edifici Cellex, Hospital Vall d'HebronBarcelona, Spain; ^2^Peptomyc, Edifici Cellex, Hospital Vall d'HebronBarcelona, Spain; ^3^Institució Catalana de Recerca i Estudis AvançatsBarcelona, Spain; ^4^Department of Biochemistry and Molecular Biology, Universitat Autònoma de BarcelonaBellaterra, Spain

**Keywords:** Myc, oncogene, inhibitor, therapy, Omomyc, clinical application

## Abstract

Myc is an oncogene deregulated in most—perhaps all—human cancers. Each Myc family member, c-, L-, and N-Myc, has been connected to tumor progression and maintenance. Myc is recognized as a “most wanted” target for cancer therapy, but has for many years been considered undruggable, mainly due to its nuclear localization, lack of a defined ligand binding site, and physiological function essential to the maintenance of normal tissues. The challenge of identifying a pharmacophore capable of overcoming these hurdles is reflected in the current absence of a clinically-viable Myc inhibitor. The first attempts to inhibit Myc used antisense technology some three decades ago, followed by small molecule inhibitors discovered through “classical” compound library screens. Notable breakthroughs proving the feasibility of systemic Myc inhibition were made with the Myc dominant negative mutant Omomyc, showing both the great promise in targeting this infamous oncogene for cancer treatment as well as allaying fears about the deleterious side effects that Myc inhibition might have on normal proliferating tissues. During this time many other strategies have appeared in an attempt to drug the undruggable, including direct and indirect targeting, knockdown, protein/protein and DNA interaction inhibitors, and translation and expression regulation. The inhibitors range from traditional small molecules to natural chemicals, to RNA and antisense, to peptides and miniproteins. Here, we briefly describe the many approaches taken so far, with a particular focus on their potential clinical applicability.

## Introduction

The Myc oncoproteins are a family of pleiotropic transcription factors that control several cellular functions related to efficient proliferation, growth and metabolism, as well as programs of tissue remodeling and regeneration (Dang, 2013). In a normal physiological context, the level of Myc proteins (comprising c-, L-, and N-Myc) is tightly regulated (Meyer and Penn, 2008; Conacci-Sorrell et al., 2014). Indeed, *myc* gene expression normally depends on growth factor signaling and both *myc* mRNA and Myc protein have very short half-lives (of 30 and 20 min respectively) (Dang, 2012). In tumor cells however, the cellular levels of Myc become independent from such signaling and regulation, and the resulting exacerbated Myc function drives intracellular and extracellular transcription programs that allow tumors to grow and thrive (Soucek and Evan, 2002; Dang, 2012; Whitfield and Soucek, 2012; Conacci-Sorrell et al., 2014; Fletcher and Prochownik, 2015). In this pathological condition, Myc deregulation can occur at any given stage of its expression (Meyer and Penn, 2008; Conacci-Sorrell et al., 2014). First, the *myc* gene itself is often subject to amplification, viral insertional events, or chromosomal translocations that provoke its exaggerated expression. Second, *myc* mRNA can become stabilized through both direct and indirect regulatory events. Third, the Myc protein turnover rate, which is normally dependent on Myc's phosphorylation status and on signaling from FBW7 to engage the ubiquitin-proteasome system, is also found altered in cancer. Finally, even when Myc is not itself mutated, its aberrant expression can occur as a consequence of upstream oncogenic signals (i.e., Ras, PI3K, Wnt, etc.) that converge on this central downstream node inside the nucleus (Meyer and Penn, 2008).

Myc functions within a network of similar proteins, called bHLH-Zip proteins, that all share a DNA-binding basic region and a bHLH-Zip dimerization domain. In this network, Myc forms heterodimers with its natural partner Max, recognizing DNA binding sites called E-boxes and thereby modulating the transcription of specific target genes (Meyer and Penn, 2008; Fletcher and Prochownik, 2015).

Given its crucial role in cancer progression and maintenance (Meyer and Penn, 2008; Dang, 2012; Hartl, 2016), Myc constitutes an ideal cancer target. However, no Myc inhibitor has reached the clinic yet, due in part to the general dogma that dominated the field for a long time claiming that Myc inhibition would cause catastrophic side effects in normal tissues, as well as to various technical issues. These include targeting a nuclear transcription factor displaying a predominantly intrinsically disordered structure, and notably, lacking a binding pocket that has been the typical target for traditional drug discovery approaches using small molecule inhibitor libraries. These issues have been addressed in recent years (Soucek et al., 2008; Prochownik and Vogt, 2010; McKeown and Bradner, 2014; Fletcher and Prochownik, 2015) and we are now witnessing a renewed interest in making Myc inhibition soon a reality for cancer patients. The technical difficulties with targeting Myc help explain the diversity of strategies that have been developed.

Recent reviews have focused on particular aspects of Myc inhibition or specific diseases (Fletcher and Prochownik, 2015; Li et al., 2015; Abedin et al., 2016; Koh et al., 2016; Posternak and Cole, 2016; Shalaby and Grotzer, 2016). Here we have given a concise overview of the strategies employed to inhibit Myc to date, with a particular focus on their applicability in clinical practice.

### Direct inhibition of Myc expression

**Figure 1:**
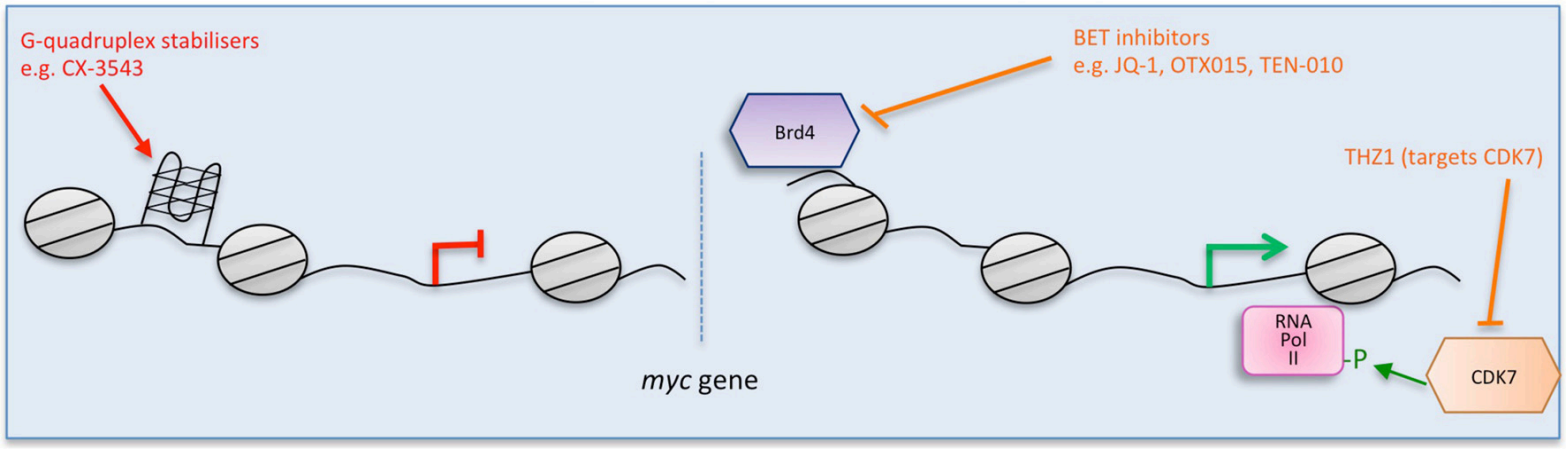
**Multiple strategies to target Myc: impairing *myc* transcription**. Direct (red) and indirect (orange) inhibitors are shown related to how they interfere with *myc*. Some examples of each inhibitor are listed. Figure adapted from Koh et al. (2016).

**Figure 2:**
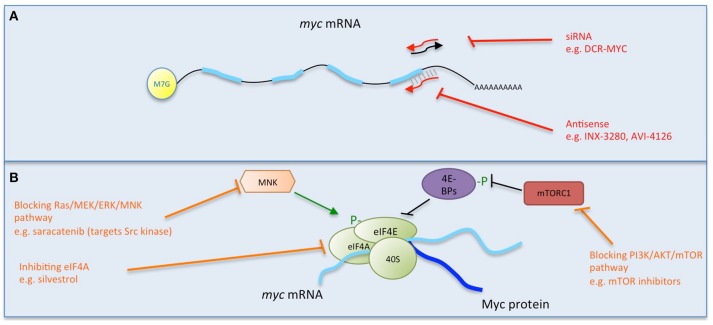
**Multiple strategies to target Myc: interfering with *myc* mRNA**. Direct (red) and indirect (orange) inhibitors are shown related to how they interfere with *myc* mRNA. **(A)** Causing the degradation of *myc* mRNA. **(B)** Preventing *myc* translation. Some examples of each inhibitor strategy are listed. Figure adapted from Koh et al. (2016).

**Figure 3:**
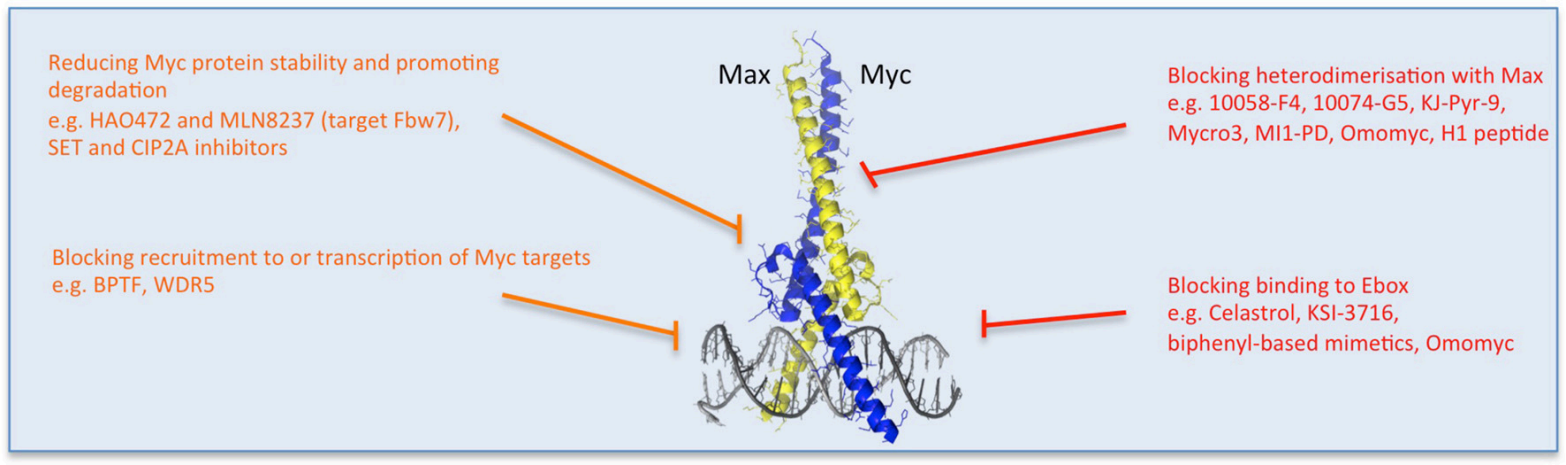
**Multiple strategies to target Myc: reducing Myc stability and function**. Direct (red) and indirect (orange) inhibitors are shown related to how they affect Myc's stability or binding to its partners or DNA. Other approaches impede Myc-dependent transcription of target genes. Some examples of each inhibitor strategy are listed. Myc/Max crystal structure is from Nair and Burley (2003) and drawn using the PyMOL Molecular Graphics System (Version 1.8 Schrodinger, LLC.).

Direct Myc inhibition can be achieved either by interference with its production or function. In the first case, one could, for example, target its transcription—either interfering with promoter accessibility and/or recruitment of transcription factors—or translation (Figures 1, 2). In the second case, efforts would likely be directed instead to preventing Myc interaction with its “partner in crime” Max or its DNA recognition binding site (Figure 3). The following section describes direct inhibitors of Myc production, while indirect inhibitors of its expression are discussed later. Table 1 provides a summary of the strategies and molecules discussed in this review.

**Table 1:** **Multiple strategies to target Myc in cancer**.

**Strategy**	**Mechanism**	**Examples**	**Preclinical/Clinical stage**	**References**
Direct inhibition of Myc expression	G-quadruplex stabilizers (prevent *myc* transcription)	CX-3543 (Quarfloxin)	Phase II in 2008	Brooks and Hurley, 2010; Drygin et al., 2011
		cationic porphyrins, quindolines, platinum complexes, ellipticine	Effective in cells	Ou et al., 2007; Pivetta et al., 2008; Wu et al., 2009; Brown et al., 2011
	Antisense oligonucleotides (prevent *myc* translation)	INX-3280	Phase I/II (discontinued)	Webb et al., 2004
		AVI-4126 (Resten-NG)	Phase I/II positive data	Devi et al., 2005; Kipshidze et al., 2003, 2004, 2007
	siRNA, microRNA (prevent *myc* translation)	DCR-MYC	Phase I/II (discontinued)	Tolcher et al., 2015
		siRNA incorporated into nanoparticles	Effective in mouse models	Conde et al., 2013; Zhu et al., 2013; Zhang et al., 2013
		siRNA in oncolytic viruses	Effective in mouse models	Li et al., 2013
Direct inhibitors of Myc that act by interfering with protein/protein interaction or binding to DNA	Small molecule protein/protein interaction inhibitors (interfere with Myc transciptional activation)	10058-F4, 10074-G5, JY-3-094, 3jc48-3	Effective in cells	Yin et al., 2003; Yap et al., 2013; Wang et al., 2013; Chauhan et al., 2014
		Mycro3, KJ-Pyr-9, MI1-PD	Effective in mouse models	Stellas et al., 2014; Hart et al., 2014; Soodgupta et al., 2015
	Compounds that specifically inhibit Myc binding to DNA (interfere with Myc transciptional activation)	KSI-3716	Effective in mouse models	Jeong et al., 2014; Seo et al., 2014
	Miniproteins or protein domains (interfere with Myc function)	Omomyc	Preclinical	Soucek et al., 2004, 2008; Sodir et al., 2011; Soucek et al., 2013; Annibali et al., 2014; Galardi et al., 2016
		H1 peptide	Effective in mouse models	Li et al., 2016; Bidwell et al., 2012, 2013
Indirect inhibition of Myc	BET bromodomain and extra-terminal domain inhibitors (may prevent *myc* transcription)	TEN-010	Phase I/II	Shapiro, 2015
		OTX015	Phase I/II	Berthon et al., 2016
		CPI-0160, ABBV-075, INCB054329, GSK525762, FT-1101	Multiple Phase I/II	Abedin et al., 2016
	Block *myc* transcription	THZ1and 2 (CDK7 inhibitors)	Effective in mouse models	Chipumuro et al., 2014; Wang et al., 2015c
	Block *myc* mRNA translation	saracatinib (Src kinase inhibitor)	Phase II	Jain et al., 2015
		mTOR/mTORCl/2 kinase inhibitors	Approved for use	Polivka and Janku, 2014; Roohi and Hojjat-Farsangi, 2016
	Target regulators of Myc protein stability	MLN8237 (Aurora-A inhibitor)	Phase II/III	Macarulla et al., 2010; Brockmann et al., 2013
		SET & CIP2A inhibitors	Preclinical	Farrell et al., 2014; Janghorban et al., 2014
Indirect targeting by synthetic lethality	Target proteins and pathways not directly related to Myc that are lethal when combined with deregulated Myc	e.g. CHK1/2, PIM and Aurora kinase inhibitors, CDK inhibitors, SAE, Pol I etc.	Numerous trials	Li et al., 2015; Cermelli et al., 2014
Indirect targeting by immunotherapy	Target immune components required for Myc-driven tumors	PCI-32765 (lbrutinib)	Multiple Phase I/II	Smith, 2015; Massó-Vallés et al., 2016
	Target immune checkpoints that are altered in Myc-driven tumors	PD-L1/CD47 inhibitors	Numerous trials and approved drugs	Casey et al., 2016

*Indirect inhibitors are those that act on a protein involved in Myc expression or function, while direct inhibitors act directly on Myc itself. The inhibitors of myc translation or transcription, for example, can be direct (acting on myc mRNA) or indirect (acting on proteins that regulate the translation or transcription of myc). The mechanism of each strategy is briefly described and examples are provided along with the stage of clinical development, if known*.

#### G-quadruplex stabilizers

G-quadruplexes (also known as G4-DNA) are tertiary structures formed in nucleic acids by sequences that are rich in guanine. The purine-rich strand in the NHE III(1) region of the Myc promoter forms G-quadruplexes (Simonsson et al., 1998, Figure 1). A number of small molecule ligands, including cationic porphyrins (Ou et al., 2007), quindolines (Pivetta et al., 2008), platinum complexes (Wu et al., 2009), and ellipticine (Brown et al., 2011) were shown to stabilize such G-quadruplexes in the *myc* gene, thus repressing its transcription. Notably, CX-3543 (Quarfloxin) was initally selected as a binder of the *myc* G-quadruplex (Chen et al., 2014) and is the only such G-quadruplex stabilizer to have reached clinical trials (entering Phase II trials for neuro-endocrine carcinomas in 2008). However, it was also shown to function by disrupting nucleolin bound to the G-quadruplexes in ribosomal DNA (Brooks and Hurley, 2009, 2010; Neidle, 2016). Since nucleolin binds to *myc* G-quadruplexes, Quarfloxin thus may also repress *myc* expression by a more indirect route (Brooks and Hurley, 2009). Development was discontinued by Cylene, although Quarfloxin has been licensed to TetraGene so development may continue.

Whether target specificity can be achieved using this strategy is not clear yet, although attempts are being made to selectively target *myc* (Felsenstein et al., 2016). According to many, total selectivity may not be necessary as long as the major target is a driver or provides a required function for the cancer cell, as for Myc (Neidle, 2016). Of course, as for some of the other types of inhibitors described here, the extent and severity of off-target side effects are key.

#### Antisense oligonucleotides

With the initial excitement over the promise of antisense as a tool to promote degradation of target mRNA, *myc* was first successfully attacked *in vitro* in multiple cell lines (Prochownik et al., 1988; Sklar et al., 1991, Figure 2A). Following these first successes, INX-3280, a 15-mer phosphorothioate oligonucleotide against the *c-myc* oncogene, was in Phase I and II clinical trials for the treatment of lymphoma and solid tumors more than a decade ago, but was discontinued in 2002 by Inex. A modified form incorporating a “transmembrane carrier system,” INXC-6295, was abandoned due to resource constraints.

With a slightly different strategy, AVI BioPharma (now Sarepta Therapeutics) developed AVI-4126 (Resten-RG), a phosphorodiamidate morpholino antisense oligomer (PMO) that inhibits Myc expression by preventing ribosomal assembly, thereby preventing mRNA translation (Arora et al., 2000). PMO is a modification shown to improve *in vivo* stability and bioavailabilty of the compound. A Phase I trial of Resten-NG carried out by AVI BioPharma enabled the determination of its bioavailability in solid tumor patients and established the feasibility of using PMOs in human cancer (Devi et al., 2005). Moreover, local delivery was feasible and safe in a related target disease, cardiovascular restenosis that involves neointimal hyperplasia (Kipshidze et al., 2003, 2004) and a Phase II study reported positive results following local delivery (Kipshidze et al., 2007; Philipp et al., 2012).

However, none of these drugs was further developed to reach the market and in fact very few antisense oligonucleotides have done so (Moreno and Pego, 2014). It is not clear though why the promising *myc* antisense approaches were not followed up, particularly in cancer studies and with newer nanocarrier delivery systems.

#### siRNA

Another approach to directly inhibit Myc expression has been used successfully *in vitro* by lentiviral delivery of shRNA (Wang et al., 2008, Figure 2A) and also attempted in clinical trials using a lipid nanoparticle formulation to deliver *myc* RNAi (DCR-MYC). While preliminary trials provided evidence of destruction of *myc* RNA in patients and a clinical response (Tolcher et al., 2015), later trial data did not meet the company's expectations for level of knockdown or efficacy, and Dicerna has halted its development.

As a relatively poor pharmacokinetic profile seems to be a limiting development factor, many attempts are being made to overcome the rapid degradation of siRNA by its incorporation into nanoparticles. For example, gold particles modified with branched polyethyleneimine have been used as efficient and non-toxic intracellular delivery agents for *c-myc* siRNA both *in vitro* (Shaat et al., 2016) and *in vivo*, where they reduced lung tumor growth after intratracheal instillation (Conde et al., 2013). Similarly, folate nanoliposomes carrying siRNA targeting *N-myc* induce tumor cell apoptosis in a neuroblastoma model *in vivo* (Zhu et al., 2013), and lipid/calcium/phosphate nanoparticles combining *c-myc* siRNA and gemcitabine into a single nanovesicle were shown to inhibit lung tumor growth with little toxicity after systemic administration in xenograft models (Zhang et al., 2013).

A related approach making use of oncolytic viruses has also been used to successfully deliver *N-myc* siRNA *in vivo* to inhibit xenograft neuroblastoma tumor growth (Li et al., 2013). Oncolytic viruses are starting to show great promise in cancer treatment (Patel and Kratzke, 2013; Andtbacka et al., 2015).

### Direct inhibitors that act by interfering with protein/protein interaction or binding to DNA

One reason for Myc's undruggability is its intrinsically disordered nature and the fact that protein interactions occur on large, flat, structurally indistinct interfaces (Prochownik and Vogt, 2010; McKeown and Bradner, 2014). However, binding to its obligate partner Max and specific DNA recognition were shown to stabilize folded conformations of Myc's bHLH-Zip domain which, despite lacking a genuine binding pocket, could constitute a relevant target for specific inhibitors. In searching for such inhibitors, the initial yeast 2-hybrid screens and subsequent FRET and fluorescence polarization assays enabled the identification of some useful small molecule compounds that established the feasibility of inhibiting Myc/Max dimers (Yin et al., 2003; Prochownik and Vogt, 2010). Since then, though, the poor bioavailability and lack of selectivity of these for Myc has limited their use *in vivo* (Prochownik and Vogt, 2010; Fletcher and Prochownik, 2015).

More recently, a surface plasmon resonance (SPR) based technique to quantitate Myc/Max interaction with a DNA probe revealed that the small molecule inhibitors described below comprise 2 distinct classes that either inhibit DNA binding by disrupting Myc/Max interaction, or by distorting the pre-formed heterodimer (Wang et al., 2015a, Figure 3).

#### Small molecule protein/protein interaction inhibitors

The peptide mimetic compound IIA6B17 was the first identified small molecule inhibitor of Myc/Max dimerization (Berg et al., 2002). Unfortunately, the activity of IIA6B17 also extended to c-Jun (Berg et al., 2002), as did the inhibitory effect of NY2267 (Xu et al., 2006), likely due to their similar structural features in their leucine zipper.

Another compound identified early on to affect Myc/Max interaction was 10058-F4 (Yin et al., 2003). Chemical modifications of 10058-F4 resulted in improvements in efficacy *in vitro*, generally correlating with their ability to disrupt Myc/Max association and DNA binding. 10058-F4 and its active analogs bind specifically to monomeric Myc, interacting with the H2/leucine zipper domain with a K_D_ of 42 μM (Wang et al., 2007). Another small molecule arising from the same screen, 10074-G5, has a K_D_ of 20 μM *in vitro* and binds to a distinct region of Myc, the basic region/H1 domain (Yin et al., 2003). Binding of both these drugs to intrinsically disordered Myc limits its ability to adopt a more defined conformation and prevents interaction with Max (Follis et al., 2008). However, both 10058-F4 and 10074-G5 are rapidly metabolized and showed poor tumor distribution, limiting their applicability *in vivo* (Guo et al., 2009; Clausen et al., 2010; Fletcher and Prochownik, 2015).

In fact, the therapeutic utility of potent small molecule inhibitors of Myc/Max dimerization has so far been limited by poor bioavailability, rapid metabolism, and inadequate target site penetration. Nevertheless, development of these small molecules continues in the hope of improving their *in vivo* characteristics (for a summary, see Fletcher and Prochownik, 2015). For example, structure-activity relationship studies of 10074-G5 led to the generation of an analog, JY-3-094, showing higher ability to disrupt the association between recombinant Myc and Max protein (Wang et al., 2013; Yap et al., 2013), but did not solve the issue of poor cell penetration. Esterification of a critical para-carboxylic acid enhanced cellular uptake, although unfortunately it impaired the ability to disrupt Myc/Max association *in vitro* (Wang et al., 2013).

Also related to 10074-G5 is the small molecule 3jc48-3, a congener that shows increased potency and stability in cell-based assays (Chauhan et al., 2014). More exciting still is Mycro3, for which daily treatment by oral gavage increased survival in a mouse model of pancreatic ductal carcinoma and orthotopic xenografts of human pancreatic cancer cells (Stellas et al., 2014). Finally, KJ-Pyr-9 is a new inhibitor found in a Kröhnke pyridine library, with a notably lower K_D_ of 6.5 nM (Hart et al., 2014). *In vivo*, KJ-Pyr-9 effectively blocks the growth of Myc-amplified human cancer cell xenografts (Hart et al., 2014). Furthermore, it penetrates the blood-brain barrier and is not acutely toxic at doses as high as 10 mg/kg (Hart et al., 2014).

New small molecules that target Myc are also being generated thanks to novel computational techniques able to virtually screen binding to different intrinsically disordered protein conformations, maximizing the chances of structure-based drug discovery. Four compounds (with the prefix PKUMDL) show micromolar affinity for Myc and activity in cell-based assays (Yu et al., 2016).

An additional “reverse” approach involves stabilizing instead the Max/Max homodimers, thus preventing Myc/Max transactivating dimers from forming. A virtual ligand screen identified a potent stabilizer of the homodimer (NSC13728) that interferes with Myc-induced transformation and transcriptional activation (Jiang et al., 2009).

In addition, attempts are being made to incorporate the small molecules into nanoparticles for increased stability and targeted delivery. For example, an Sn2 lipase-labile pro-drug inhibitor (MI1-PD) conjugated to integrin-targeted nanoparticles extended survival in a mouse model of multiple myeloma (Soodgupta et al., 2015).

A more extensive review of these small molecule inhibitors is provided elsewhere (Chen et al., 2014). Such inhibitors have been an intense focus in Myc inhibition research for many years, and we hope that further preclinical development of promising leads is ongoing.

#### Compounds that specifically inhibit Myc binding to DNA

Other small molecule inhibitors such as MYRA-A and NSC308848 have achieved high selectivity in targeting the DNA-binding domain of Myc/Max, and preventing specific interaction with DNA (Mo and Henriksson, 2006; Mo et al., 2006, Figure 3).

Some naturally-occurring molecules have also been shown to directly interact with Myc/Max heterodimers. Celastrol and celastrol-inspired triterpenoids bind to and alter the quaternary structure of the pre-formed dimer and abrogate its DNA binding (Wang et al., 2015b).

KSI-3716 also blocks Myc/Max binding to DNA, and inhibited orthotopic tumor formation after local instillation to the bladder (Jeong et al., 2014)—the typical treatment route for this cancer type—even in gemcitabine-resistant tumors (Seo et al., 2014).

As mentioned above, though, small molecules, despite good affinity for their target *in vitro*, often display lack of selectivity in cells or *in vivo*. In order to try to overcome this issue, synthetic α-Helix “mimetics” based on biphenyl were developed (Jung et al., 2015). These have an increased interaction surface and recognize Myc/Max dimers (not free Myc) and disrupt DNA binding. However, their K_D_ was not increased (13 μM) and specificity was not enhanced: similar activity was observed in non-cancer cells lacking Myc overexpression (Jung et al., 2015).

#### Miniproteins or protein domains

Miniproteins or protein domains comprise a group of structurally-related molecules based on domains from Myc family members (Figure 3). The best characterized so far and especially notable for its *in vivo* use, is Omomyc. Omomyc has been well validated as a gene therapy, providing the proof of concept for the feasibility of systemic Myc inhibition. It comprises the bHLH-Zip domain of Myc carrying four aminoacidic substitutions that alter its dimerization specificity, such that in addition to binding Myc's natural partner Max, it can also heterodimerize with Myc as well as homodimerize (Soucek et al., 1998, 2002; Savino et al., 2011). As a result, Omomyc acts as a dominant negative of Myc transcriptional function, being able to disrupt Myc/Max interaction, sequester Myc away from DNA and occupy the E-box with transcriptionally inactive dimers (Omomyc/Omomyc and/or Omomyc/Max). Notably, in doing so, it antagonizes all Myc family members (Soucek et al., 2008; Savino et al., 2011; Fiorentino et al., 2016). Multiple studies in mouse models of cancer demonstrated Omomyc's therapeutic impact in different types of cancer, independently of their driving mutation or tissue of origin, pointing to the key role of Myc in tumorigenesis downstream of the diverse oncogenic lesions (Soucek et al., 2004, 2008, 2013; Sodir et al., 2011; Annibali et al., 2014; Galardi et al., 2016). Importantly, in each model Omomyc showed only minimal side effects, suggesting its safety and potential applicability in patients. Work to translate its use from gene therapy to pharmacological application is currently ongoing (Peptomyc S.L.).

Another modified Myc peptide, this time a 14 amino acid sequence from the helix 1 (H1) carboxylic region of Myc harboring 2 changes, was shown to be active against breast cancer cells in culture when fused to a fragment from *Antennapedia* to enable cellular uptake (Giorello et al., 1998). This has not performed well *in vivo*, at least partly because it does not efficiently cross the nuclear envelope. However, recently a staggered, “dual-strike” strategy was employed, whereby a first treatment with docetaxel arrested the cells in G2/M, prolonging the period of nuclear envelope disassembly, followed by a second treatment, this time with the H1 peptide (Li et al., 2016). *In vivo*, this procedure reduced the growth of subcutaneously-inoculated HeLa cells and prolonged animal survival (Li et al., 2016). The peptide was delivered in the macromolecular carrier HPMA.

The H1 peptide was also used for an *in vivo* study by fusing it to both a cell-penetrating peptide sequence and an elastin-like polypeptide (ELP) that is thermally stable and allows targeted delivery to particular tissues by local hyperthermia (Bidwell et al., 2013). This multi-functional peptide reduced tumor growth in rodent orthotopic models of glioma and breast cancer (Bidwell et al., 2012, 2013).

Another protein domain that could be used to inhibit Myc is the bHLH-Zip of Max (Montagne et al., 2012). This truncated protein spontaneously transduces into cells and inhibits Myc transcription. The idea behind this strategy would be to provide excess homodimeric Max to out-compete Myc/Max heterodimers binding to DNA.

### Indirect inhibition of Myc

Targeting Myc itself has often proven very challenging. Because of that, many researchers have instead opted for an indirect approach, focusing on Myc transcriptional regulation or modulation of stability and activity, by inhibiting more tractable targets and not directly hitting Myc itself. Here is an overview of these alternative approaches, once again encompassing the transcription, translation and stability of Myc, as well as controlling its activity as a transcription factor.

#### Blocking *myc* transcription

The BET bromodomain and extra-terminal domain inhibitors are a significant area of focus at the moment and were originally described to target Myc expression. A selective small molecule inhibitor (JQ1) of BET bromodomains was unexpectedly found to downregulate Myc (Delmore et al., 2011). JQ1 displaces the bromodomain chromatin regulators from the large super-enhancers of genes connected with multiple myeloma, notably *myc* (Delmore et al., 2011; Loven et al., 2013, Figure 1). JQ1 preferentially impacts on *myc* transcription, causing cell-cycle arrest, and cellular senescence, as well as resulting in significant anti-tumor activity in mouse models of multiple myeloma, and xenograft models of Burkitt's lymphoma and acute myeloid leukemia, diseases in which the *myc* gene is amplified (Delmore et al., 2011; Mertz et al., 2011).

After the initial excitement about their potential for Myc inhibition, it is now becoming clear that JQ1—and other BET inhibitors—may function by inhibiting additional oncogenic factors besides Myc (Andrieu et al., 2016), whose levels remain unaffected in some cellular contexts (Ambrosini et al., 2015; Yao et al., 2015; Bid et al., 2016; Garcia et al., 2016; Hogg et al., 2016; Donato et al., 2017). Moreover, compensatory mutations have been identified that can prevent Myc downregulation by BET inhibitors and/or their therapeutic efficacy (Shimamura et al., 2013; Fong et al., 2015; Rathert et al., 2015; Shi et al., 2016). Nevertheless, several BET inhibitors are currently in early-phase clinical trials in various hematologic malignancies (Abedin et al., 2016). Some patients in dose-escalation Phase I studies have shown complete or partial remission of acute leukemia after treatment with OTX015 (Berthon et al., 2016), and data is currently available for 3 patients treated with TEN-010 in a study of NUT-midline carcinoma showing clinical responses in both patients receiving the higher dose (Shapiro, 2015). It remains to be seen how much of the effect in patients is actually due to Myc inhibition.

An unrelated approach to blocking *myc* transcription targets CDK7, a catalytic subunit involved in the transcriptional initiation complex that phosphorylates serine-5 of RNA pol II (Nilson et al., 2015). A covalent inhibitor (THZ1) was developed that showed selectivity for CDK7, and specifically downregulated Myc in neuroblastoma (Chipumuro et al., 2014; Kwiatkowski et al., 2014). While THZ1 selectivity for Myc is unclear and it is possible that its therapeutic impact is due to other targets as well, it is effective at treating lung cancer (Christensen et al., 2014) and triple-negative breast cancer cell lines (Wang et al., 2015c) as well as N-Myc driven neuroblastoma in mice (Chipumuro et al., 2014). Furthermore, the analog THZ2 was developed that has improved pharmacokinetics (Wang et al., 2015c).

#### Blocking *myc* mRNA translation

Another approach to interfere with Myc protein expression is to block its translation (Figure 2). *myc* mRNA can be translated both by 5′ cap- and internal ribosome entry site (IRES)- dependent mechanisms (Nanbru et al., 1997). The central role of mTOR in mediating the translation of mRNAs such as *myc* via these mechanisms suggests that targeting mTOR or upstream controllers of its activity (PI3K/PTEN/AKT and Ras/Raf/MEK/ERK) could be a fruitful strategy, and there are multiple inhibitors of these pathways.

While inhibition of mTOR alone in a mouse model of colorectal tumorigenesis failed to inhibit translation of *myc*, a small molecule inhibitor of eIF4A, silvestrol, effectively reduced *myc* translation, inhibiting tumor growth (Wiegering et al., 2015). Multiple mTOR and mTORC1/2 kinase inhibitors are currently approved for clinical use, and there is a significant focus on targeting many members of these signaling pathways (Polivka and Janku, 2014; Roohi and Hojjat-Farsangi, 2016).

Again though, inhibitors of such targets will have effects not limited to *myc* alone.

Recent data also indicate that concomitant targeting of HDAC and PI3K would be beneficial to the treatment of Myc-driven tumors. In particular the use of CUDC-907, a small molecule inhibitor of both HDACs and class I PI3Ks, was shown to be effective in reducing the growth and survival of Myc-transformed cancer cells and demonstrated therapeutic impact in multiple mouse models of Myc-dependent tumors (Sun et al., 2016). CUDC-907 is currently in Phase II clinical trials to study and evaluate its efficacy and safety (alone or in combination with Rituximab) in patients with Relapsed/Refractory (RR) Myc-altered Diffuse Large B-Cell Lymphoma (DLBCL), including patients with Myc alterations.

Another recent translation inhibition approach made use of Src kinase blockade with the small molecule inhibitor saracatinib in preclinical studies in premalignant breast cells and tissue. Among other effects, saracatinib inhibited the ERK1/2-MNK1-eIF4E-mediated cap-dependent translation of *myc* (Jain et al., 2015).

#### Targeting regulators of Myc protein stability

The regulation of Myc stability is complex. Numerous studies propose the targeting of ubiquitinases or phosphatases for the degradation of Myc in cancer cells. Clearly, whether these can be specific for Myc and/or have sufficiently minimal side effects is still unclear.

Myc is ubiquitinated by a number of E3 ligases, such as SCF (FBW7) and SCF (Skp2). One approach is therefore to inhibit the deubiquitinases that help stabilize Myc, such as USP28, USP38, and USP36 (Sun et al., 2015). On the other hand, proteasomal degradation could be triggered, for example by oridonin, a natural plant diterpenoid that induces FBW7-mediated Myc breakdown (Huang et al., 2012). Oridonin derivatives are in clinical trials (e.g., HAO472 for leukemia treatment), although such anticancer drugs (and ubiquitin/deubiquitinases) have numerous other potential mechanisms of action and targets, not solely limited to Myc inhibition.

It has been shown that N-Myc complexes with the Aurora-A kinase and is thus protected from proteasomal degradation (Otto et al., 2009). Aurora-A inhibitors (MLN8054 and MLN8237) disrupt the complex and promote N-Myc degradation by FBW7 ubiquitin ligase (Brockmann et al., 2013). While clinical development of MLN8054 was terminated by Millenium in 2008 due to side effects (Macarulla et al., 2010), the more potent, selective, second generation inhibitor MLN8237 (Alisertib) is currently being evaluated in multiple Phase II and III studies (based on the obligate role of Aurora kinases in mitosis). Since N-Myc is a critical target of this class of Aurora-A inhibitors, perhaps it is possible to design allosteric inhibitors that more strongly distort Aurora-A and thereby more effectively disrupt the Aurora-A/N-Myc complex, while retaining the ability of Aurora-A to promote mitotic entry, thus finding a more specific inhibitor (Brockmann et al., 2013). Notably, the Aurora kinases have turned up in multiple screens for Myc synthetic lethal targets (see later section).

Another ubiquitin ligase—HUWE1 (HECTH9, ARF-BP1, MULE)—has also been targeted by small molecule inhibitors. These reduce Myc-dependent transactivation in colorectal cancer cells, but not in stem and normal colon epithelial cells, by influencing Myc and Miz1 degradation (Peter et al., 2014).

Alternatively, the tumor suppressor protein phosphatase 2A (PP2A) targets serine 62 of Myc and causes its inactivation and destabilization (Sears, 2004). Cellular inhibitors of PP2A (the SET oncoprotein and CIP2A, the cancerous inhibitor of PP2A) are increased in human cancers and lead to overexpression of Myc (Westermarck and Hahn, 2008). Thus, inhibitors of SET and CIP2A were used to reduce Myc expression and activity, decreasing the tumorigenic potential of cancer cells (Farrell et al., 2014; Janghorban et al., 2014). Inhibitors are in preclinical development; the effects of PP2A activation though would likely not be limited to Myc degradation alone, since other pathways would be targeted as well.

### Indirect targeting by synthetic lethality

Described as “using a back door to target Myc” (Evan, 2012), in this indirect approach, the targets are the cellular changes that arise as a consequence of oncogene activation of proteins and pathways required for survival of the oncogene-addicted cells (Wang et al., 2004). Myc-mediated synthetic lethality was first described to be induced by TRAIL and DR5-agonists, taking advantage of Myc's intrinsic ability to prime cells to apoptotic stimuli (Wang et al., 2004).

More recently, SAE1/2 was identified in a genome-wide RNA interference screen to search for Myc synthetic lethal genes. This SUMOylation enzyme is required for proper mitotic spindle function, and proved necessary for Myc-driven tumorigenesis as its inhibition caused mitotic catastrophe in Myc hyperactive cells (Kessler et al., 2012). Identification of SUMO inhibitors is ongoing (Kumar et al., 2016).

A number of studies demonstrate that inhibition of CDKs is also synthetic lethal with Myc. Pharmacological inhibition of CDK2 forces embryonic fibroblasts with deregulated Myc into senescence (Hydbring et al., 2010), while CDK2 ablation induces senescence in B-cells after Myc activation, delaying lymphomagenesis (Campaner et al., 2010). Since the function of CDK2 is compensated by other CDKs in normal cells (Ortega et al., 2003), this suggests that its selective targeting could be used therapeutically, at least in Myc-driven tumors.

CDK1 inhibition is also beneficial for treating Myc-overexpressing tumors: the CDK1 inhibitor purvalanol A induces substantial apoptosis in cells overexpressing Myc, but not in cells expressing other oncogenes (Goga et al., 2007). It prolongs survival in *E*μ*-myc* transgenic mice and lymphoma allograft models (Goga et al., 2007).

Finally, synthetic lethality was observed with CDK9. Its pharmacological inhibition or knockdown by shRNA is anti-tumorigenic both in cells and mouse models of hepatocellular carcinoma, the extent of its effect correlating with Myc levels (Huang et al., 2014).

Since several CDK inhibitors are currently in clinical trials (Lapenna and Giordano, 2009), there is clear merit in analyzing the results taking into account this potential Myc synthetic lethality.

Myc has been shown to control multiple aspects of transcription and co-transcription, as well as RNA metabolism, including splicing, mRNA stability, and translation efficiency (Koh et al., 2016). In the context of the translation machinery, Myc is able to modulate Ribosomal Biogenesis (RiBi) through the coordinated regulation of all three RNA polymerases: Pol I, Pol II, and Pol III. Selective inhibition of Pol I transcription has been proposed as a promising therapeutic approach in Myc-driven cancers (Poortinga et al., 2015), as Myc is supposed to prime cells to nucleolar stress. Remarkably, despite the risk associated to interfering with a central engine of a “housekeeping” process such as RiBi, an inhibitor of Pol I (CX-5461; Drygin et al., 2011) has recently demonstrated sufficient safety in Phase I clinical trials in patients with lymphoma and leukemia, and is now in a Phase I/II study in solid malignancies (although not confined to Myc-driven cancers only).

Myc synthetic lethality has been observed with various other targets in mouse tumor models, for example with ARK5 (Liu et al., 2012), PIM kinase (Horiuchi et al., 2016), microRNA-206 that acts by inhibiting MAP3K13 (Han et al., 2016), Aurora kinases (den Hollander et al., 2010; Yang et al., 2010), CHK1 (Murga et al., 2011; Ferrao et al., 2012), and MondoA (Carroll et al., 2015). MondoA had been previously linked to the regulation of glucose metabolism while overexpression of Myc in mammalian cells renders them addicted to certain metabolic pathways (Shim et al., 1998; Yuneva et al., 2007) thus prompting the extension of these synthetic lethal screen to genes involved in metabolism. This led to the identification of Myc synthetic lethal metabolic genes involved in glycolysis (ALDOA and PDK1) and nucleotide biosynthesis (CTPS) (Toyoshima et al., 2012); purine biosynthesis (PFAS and CAD), trans-sulphuration (CBS), mitochondrial transcription (TFAM), glycolysis (ENO3), and lipogenesis (FASN and SCD) (Carroll et al., 2015); and glutamine/glutamate (SLC1A4 and SLC25A6) (Toyoshima et al., 2012; Carroll et al., 2015).

It is well documented that Myc contributes to metabolic adaptations in cancer cells (Dang, 2012). As a proof of concept, inhibition of various metabolic targets, such as LDHA and glutaminase, reduced tumor growth, and extended survival in Myc-dependent and Myc-inducible cancer models, although the synthetic lethality with Myc was not specifically demonstrated (Hsieh and Dang, 2016).

This indirect strategy has therefore already provided a variety of additional therapeutic targets. Some clinical trials are ongoing (e.g., inhibitors of CHK1/2, PIM, and Aurora kinases). Whether these would be relevant for tumors in which Myc is not a driving oncogene has to be seen, but the fact that Myc is causally linked to most human cancers suggests that this approach warrants further clinical investigation.

### Indirect targeting by immunotherapy

This rather different approach to cancer treatment is currently receiving a significant amount of interest worldwide. One strategy would be to prime the immune system to target Myc-overexpressing tumors. As a proof of concept, immunization of mice with human Myc generated T-cells that helped protect some mice from a lethal lymphoma (Helm et al., 2013).

A more direct immunotherapy could target specific immune system components required for Myc-induced tumorigenesis, such as mast cells (Soucek et al., 2007). PCI-32765 (Ibrutinib) is an inhibitor of Bruton's tyrosine kinase (BTK) that is clinically approved for the treatment of multiple cancers (Smith, 2015; Massó-Vallés et al., 2016) and was shown to inhibit Myc-driven pancreatic islet tumor formation (Soucek et al., 2011).

Alternatively, Myc-driven tumors may downregulate the anti-tumor immune response by producing PD-L1 and CD47, two key immune checkpoints, making them eligible for treatment with immune-based therapies that target such checkpoints (Casey et al., 2016).

## Final remarks

An impressive array of strategies has been developed for drugging Myc. These take advantage of multiple mechanisms, acting both directly and indirectly, and impacting on Myc in contrasting ways (see Table 1 for a summary). Many approaches have yielded molecules that entered clinical trials.

Early clinical studies with antisense inhibitors of *myc* did not progress, nor did a recent trial with *myc* RNAi (DCR-MYC), although the approach might still be valid in some applications. In fact, incorporation of *myc* siRNA into nanoparticles is an active field of research yielding novel carrier formulations with *in vivo* efficacy. Interference with Myc translation is an approach that has clearly advanced in the past few years and various groups and companies are pursuing it.

Similarly, the initial small molecule inhibitors showed poor bioavailability, but development of these compounds and further screens have identified compounds with improved *in vivo* activity, pharmacokinetics and bioavailability, even with systemic administration (e.g., Mycro3, KJ-Pyr-9). In addition, efforts are being directed toward incorporating small molecules into nanocarriers and some are starting to show *in vivo* efficacy (MI1-PD). Published *in vivo* or clinical trial data is lacking for a number of other small molecule inhibitors and natural compounds shown to target Myc in cell culture; it seems sensible to at least determine their *in vivo* efficacy and bioavailability and one hopes that no promising leads have fallen by the wayside.

Additional strategies such as BET inhibitors and G-quadruplex stabilizers (Quarflaxin) have progressed to clinical trials, and yet more strategies are in preclinical development, such as peptides and protein domains (Omomyc).

It is important to mention that new therapeutic opportunities constantly appear, thanks to the ever-growing knowledge regarding Myc biology and function. For example, novel strategies could soon be based on the potential inhibition of co-factors that determine Myc specific recruitment to chromatin and recognition of target genes. Among these factors, WDR5 and BPTF are probably the best characterized. WDR5 is a WD40-repeat protein that functions within the context of several chromatin-regulatory complexes (Thomas et al., 2015). WDR5 interaction with Myc facilitates its recruitment to chromatin and recognition of target genes. Similarly, in the context of chromatin access, BPTF, a core subunit of the NURF chromatin-remodeling complex, has been described as a key Myc interactor that allows its recruitment to DNA targets (Richart et al., 2016a,b). Inhibition of interaction of such proteins with Myc by small molecules could offer a novel therapeutic opportunity. Once again, it is likely that such small molecules would also affect other cellular functions that require these two important epigenetic regulatory proteins, but it is worth trying to develop molecules specific for the interface with Myc and validate them in experimental models, before discarding them *a priori*.

Other future strategies could include development of viral-mediated delivery of inhibitors such as shRNA, Omomyc or even Crispr to delete *myc* in tumor cells. It is likely, however, that the most efficient Myc inhibition strategy will not be limited to a single approach, but rather a combination of targeting methods. These may include low-dose combinations of drugs that each act to reduce Myc levels in different ways (Brockmann et al., 2013) or that act on different aspects of Myc biology.

In this context, no attempt should be left aside to finally overcome the challenge of targeting the “undruggable,” because its impact in the clinic would clearly be dramatic.

Interestingly, the search for a clinically-viable Myc inhibitor has recently been likened to the hunt for the Higgs boson (Lazo and Sharlow, 2016), which was finally discovered in 2012. After the success of that 40-year search, we can be hopeful that it will not be too long before that other elusive particle—a Myc inhibitor—emerges from clinical trials.

## Author contributions

JW wrote the review with help from MB and LS.

## Funding

We gratefully receive support from Fundación BBVA, Worldwide Cancer Research (WCR/AICR Grant #13-1182), the European Research Council (CoG Grant #617473), Instituto de Salud Carlos III (FIS Grants #PI13/01705, #PI16/01224) and the FERO Foundation. MB is supported by the Fonds de Recherche du Québec en Santé.

### Conflict of interest statement

LS and MB are co-founders and shareholders of Peptomyc S.L., a spin-off company located at VHIO. The other author declare that the research was conducted in the absence of any commercial or financial relationships that could be construed as a potential conflict of interest.
